# Left ventricular assist devices: a kidney’s perspective

**DOI:** 10.1007/s10741-015-9481-z

**Published:** 2015-03-22

**Authors:** T. R. Tromp, N. de Jonge, J. A. Joles

**Affiliations:** 1 University Medical Center Utrecht, POB 85500, 3508 GA Utrecht, The Netherlands; 2 Department of Cardiology, University Medical Center Utrecht, Utrecht, The Netherlands; 3 Department of Nephrology and Hypertension, University Medical Center Utrecht, Utrecht, The Netherlands

**Keywords:** Left ventricular assist device, Renal function, Cardiorenal syndrome, Chronic kidney disease, Acute kidney injury, Mechanical circulatory support

## Abstract

The left ventricular assist device (LVAD) has become an established treatment option for patients with refractory heart failure. Many of these patients experience chronic kidney disease (CKD) due to chronic cardiorenal syndrome type II, which is often alleviated quickly following LVAD implantation. Nevertheless, reversibility of CKD remains difficult to predict. Interestingly, initial recovery of GFR appears to be transient, being followed by gradual but significant late decline. Nevertheless, GFR often remains elevated compared to preimplant status. Larger GFR increases are followed by a proportionally larger late decline. Several explanations for this gradual decline in renal function after LVAD therapy have been proposed, yet a definitive answer remains elusive. Mortality predictors of LVAD implantation are the occurrence of either postimplantation acute kidney injury (AKI) or preimplant CKD. However, patient outcomes continue to improve as LVAD therapy becomes more widespread, and adverse events including AKI appear to decline. In light of a growing destination therapy population, it is important to understand the cumulative effects of long-term LVAD support on kidney function. Additional research and passage of time are required to further unravel the intricate relationships between the LVAD and the kidney.

## Introduction

Approximately 1–2 % of the adult population in developed countries suffers from heart failure (HF) [1]. In the USA, an estimated 5.7 million people suffer from HF [2], whereas worldwide the number of HF patients exceeds 23 million [3]. Although most cases can be managed pharmacologically and/or surgically, HF may progress and become unresponsive to conventional treatment [4]. For these refractory HF patients, encompassing an estimated 5–10 % of the total HF population [5], heart transplantation (HTx) is currently the gold standard of treatment [4, 6–8]. However, HTx is limited by availability of donor hearts [7] and patients may not always meet criteria for placement on waiting lists [4]. Eurotransplant reported an increasing number of patients waiting for HTx, a trend unmatched by donor heart availability [9]. Consequently, waiting list mortality remains too high [10, 11].

Implantable left ventricular assist devices (LVAD) have revolutionized treatment of late-stage systolic HF [7, 12]. An LVAD is an implantable mechanical circulatory support (MCS) device, powered by an external driveline cable, which aids the failing heart by unloading the left (or right) ventricle. In 2001, the pivotal REMATCH trial showed that LVAD therapy was superior to maximal medical therapy: 1-year survival rate of the LVAD group doubled that of the control group receiving such therapy (52 vs. 25 %) [13]. Although LVADs were first accepted to support patients awaiting HTx, the so-called bridge to transplantation therapy, they are now increasingly being offered to patients ineligible for HTx. Such destination therapy (DT) can be seen as an alternative to HTx [14, 15]. It has to be noted that some patients initially intended for DT may improve sufficiently to become HTx eligible again, the bridge to candidacy population. This implies that the division between bridge to transplantation therapy and DT is not always entirely black and white.

The first-generation LVAD pumps were large and pneumatically driven, creating pulsatile-flow (pf). However, these devices showed many adverse events. The new generation of continuous-flow (cf) pumps is smaller, more durable and shows a considerably improved safety profile [16]. Moreover, cf-LVADs are easier to implant, operate silently, but create high shear stress and areas of stasis [17]. Retrospective analysis of large patient samples has shown that cf-LVADs offer superior survival over pf-LVADs with fewer adverse events [16] and at lower cost [18]. However, the non-physiologic nature of these devices has been topic of debate. Since 2010, continuous-flow devices accounted for over 99 % of LVADs implanted in the USA [16].

Many LVAD patients experience renal impairment secondary to HF, prior to pump placement. Baseline eGFR, at the time of LVAD implantation, averages 60 (±35) mL/min/1.73 m^2^ [19]. HF combined with chronic kidney disease (CKD) is significant public health problems with increasing overlap [20]. Two out of three patients hospitalized for HF also present with CKD [21], defined as estimated creatinine clearance <60 mL/min/1.73 m^2^. Cardiorenal syndrome (CRS) refers to a group of acute and chronic clinical conditions in which failure of either heart or kidney initiates or aggravates failure of the other organ [22]. Increased efforts are directed toward classification, identification and understanding of the pathogenesis of combined heart and kidney diseases [23]. The subtypes of CRS are categorized depending on primary organ dysfunction and acute versus chronic onset [24], as shown in Table 1. However, it should be noted that the validity of this classification is under debate [22].

**Table 1:** Classification of cardiorenal syndromes

Cardiorenal syndrome (CRS) general definition A pathophysiologic disorder of the heart and kidneys whereby acute or chronic dysfunction in one organ may induce acute or chronic dysfunction in the other organ
CRS type I (acute cardiorenal syndrome) Abrupt worsening of cardiac function (e.g., acute cardiogenic shock or acutely decompensated congestive heart failure) leading to acute kidney injury
CRS type II (chronic cardiorenal syndrome) Chronic abnormalities in cardiac function (e.g., chronic congestive heart failure) causing progressive and potentially permanent chronic kidney disease
CRS type III (acute renocardiac syndrome) Abrupt worsening of renal function (e.g., acute kidney ischemia or glomerulonephritis) causing acute cardiac disorder [e.g., heart failure, arrhythmia, ischemia)
CRS type IV (chronic renocardiac syndrome) Chronic kidney disease (e.g., chronic glomerular or interstitial disease) contributing to decreased cardiac function, cardiac hypertrophy and/or increased risk of adverse cardiovascular events
CRS type V (secondary cardiorenal syndrome) Systemic condition (e.g., diabetes mellitus, sepsis) causing both cardiac and renal dysfunction

Adapted from McCullough et al. [24]

## Cardiorenal syndrome type II

**Fig. 1:**
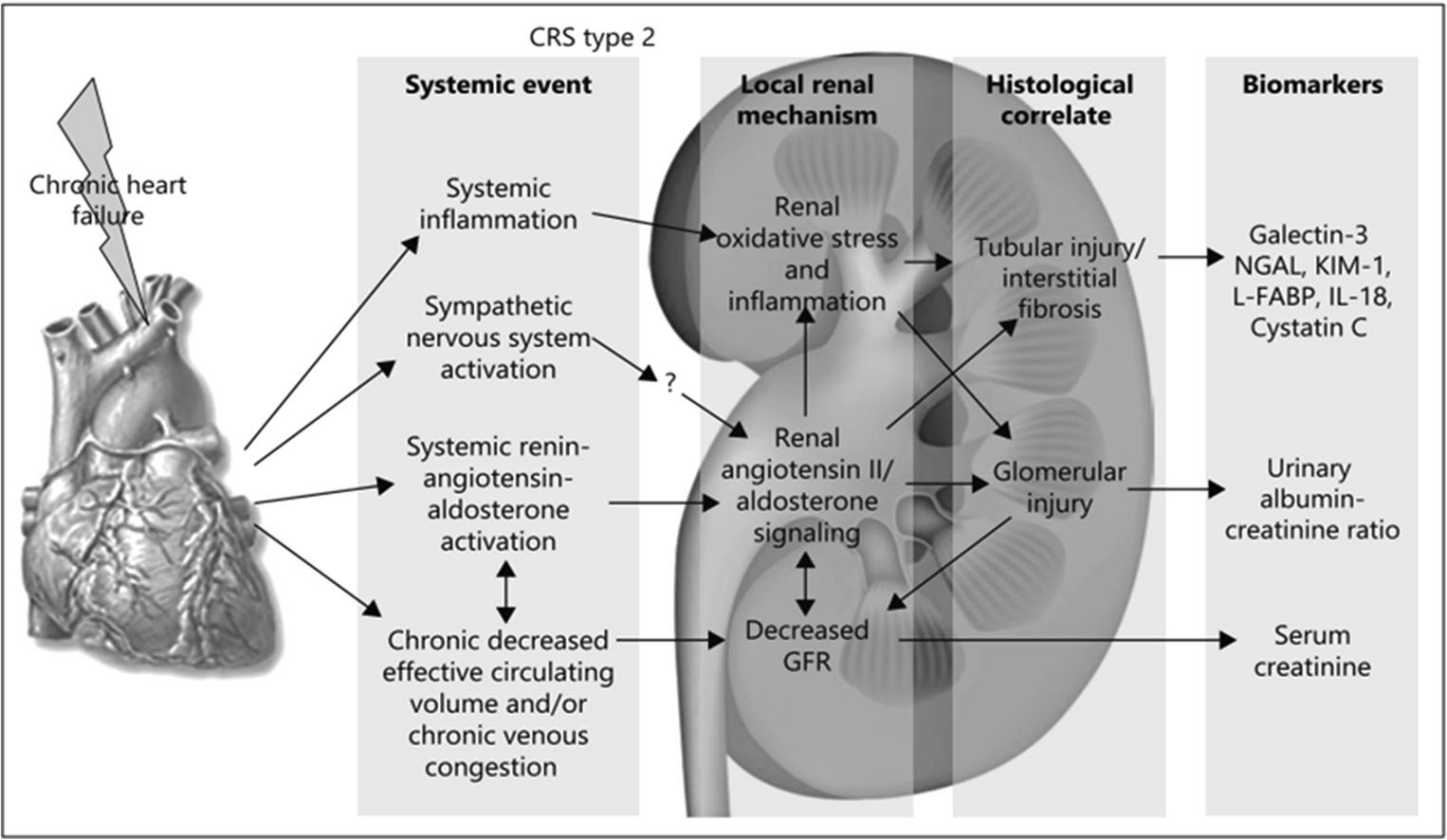
Pathophysiology of CRS type II (reprinted with permission [25] ). NGAL, neutrophil gelatinase-associated lipocalin; KIM 1, kidney injury molecule-1; L-FABP, liver-type fatty acid binding protein; IL-18, interleukin-18

The fact that many patients hospitalized for HF also present with CKD can mainly be explained by the pathophysiology of CRS type II. The pathophysiology of chronic CRS type II (CKD on top of HF) is largely derived from animal studies since it is difficult to exclude confounding factors and establish temporal relationships in humans [25]. Proposed pathophysiological mechanisms include neurohormonal activation, hemodynamic factors such as renal hypoperfusion and venous congestion, inflammation and oxidative stress [25]; mechanisms are summarized in Fig. 1. In CRS type II, chronic abnormalities in cardiac function can cause progressive and permanent kidney injury [25].

The first established cardiorenal connectors were hemodynamic factors [22]. Significant increases in renal venous pressure are transmitted to intratubular pressure, which directly decreases net filtration pressure, thereby diminishing GFR. Venous congestion resulting from inadequate left ventricular output has been reported as the most important hemodynamic factor resulting in worsening renal function in advanced HF patients [24–27].

However, chronic CRS type II cannot only be explained by hemodynamic factors [22]. Falling cardiac output and reduced renal perfusion results in activation of both the sympathetic nervous system and the renin angiotensin aldosterone system (RAAS) [28]. It has been shown that renal venous hypertension can induce RAAS activation, independent of changes in systolic blood pressure (BP) and flow [25]. Water and sodium retention resulting from RAAS activation will increase fluid volume and thus workload of the already faltering heart. Heart and kidney can subsequently enter a vicious circle, inevitably leading to decompensated HF. It has been proposed that activation of the sympathetic nervous system and local angiotensin II stimulates NADPH oxidase-dependent reactive oxygen species generation in the kidney, leading to podocyte injury and albuminuria [29]. Moreover, paracrine aldosterone signaling can provoke oxidative stress, which can lead to renal fibrosis [25].

Another non-hemodynamic factor contributing to CKD in HF patients is an inflammatory response in the kidneys. Cardiac monocytes, under stress of mechanical stretch or ischemia, can produce pro-inflammatory cytokines that may have distant effects on the kidneys [25]. In addition, venous congestion may precipitate intestinal ischemia, enhancing translocation of intestinal endotoxin-containing bacteria into the bloodstream [30], leading to a pro-inflammatory state [25].

There has been increasing interest in new biomarkers such as NGAL, KIM-1 and L-FABP (depicted in Fig. 1) to assess and predict renal injury [31, 32]. However, these biomarkers are not (yet) in routine use in the clinic and will therefore not be further explored in this review.

## Preimplant renal function and survival

Baseline eGFR prior to LVAD implantation averages 60 (±35) mL/min/1.73 m^2^ [19]. Severity of preimplant renal dysfunction is inversely related to postimplant survival [16]. Kirklin et al. [33] retrospectively analyzed the Interagency Registry for Mechanical Circulatory Support (INTERMACS) database and concluded that preimplant renal dysfunction (RD) predicts higher mortality after LVAD-implant. This has been confirmed by studies in Europe and Asia [34, 35]. In a study not limited to LVAD patients, Hillege et al. [36] drew a similar conclusion: RD in HF patients predicts longer hospitalization and worse long-term survival outcomes. This reduced survival of patients with severe preimplant RD is most pronounced in the early postoperative course: After the initial postoperative period, death rates appear independent of preimplant RD [33].

Since preimplant RD increases early postimplant mortality, it is imperative that patients receive timely referral for LVAD therapy, before HF worsens and leads to CKD [37]. In fact, those patients with marked RD tend to represent the old and very sick; subgroups with inherent decreased perioperative survival [38].

## Predictor models

In predicting perioperative mortality, renal parameters are incorporated in various risk scoring systems [39–42]. As a general rule, RD and high diuretic doses, among others, serve as poor prognostic markers [38]. It has recently been proposed that AKI after implantation serves as a more reliable predictor of mortality in this patient population [43, 44]. This warrants close monitoring of immediate postoperative renal function and minimization of modifiable risk factors for AKI.

**Fig. 2:**
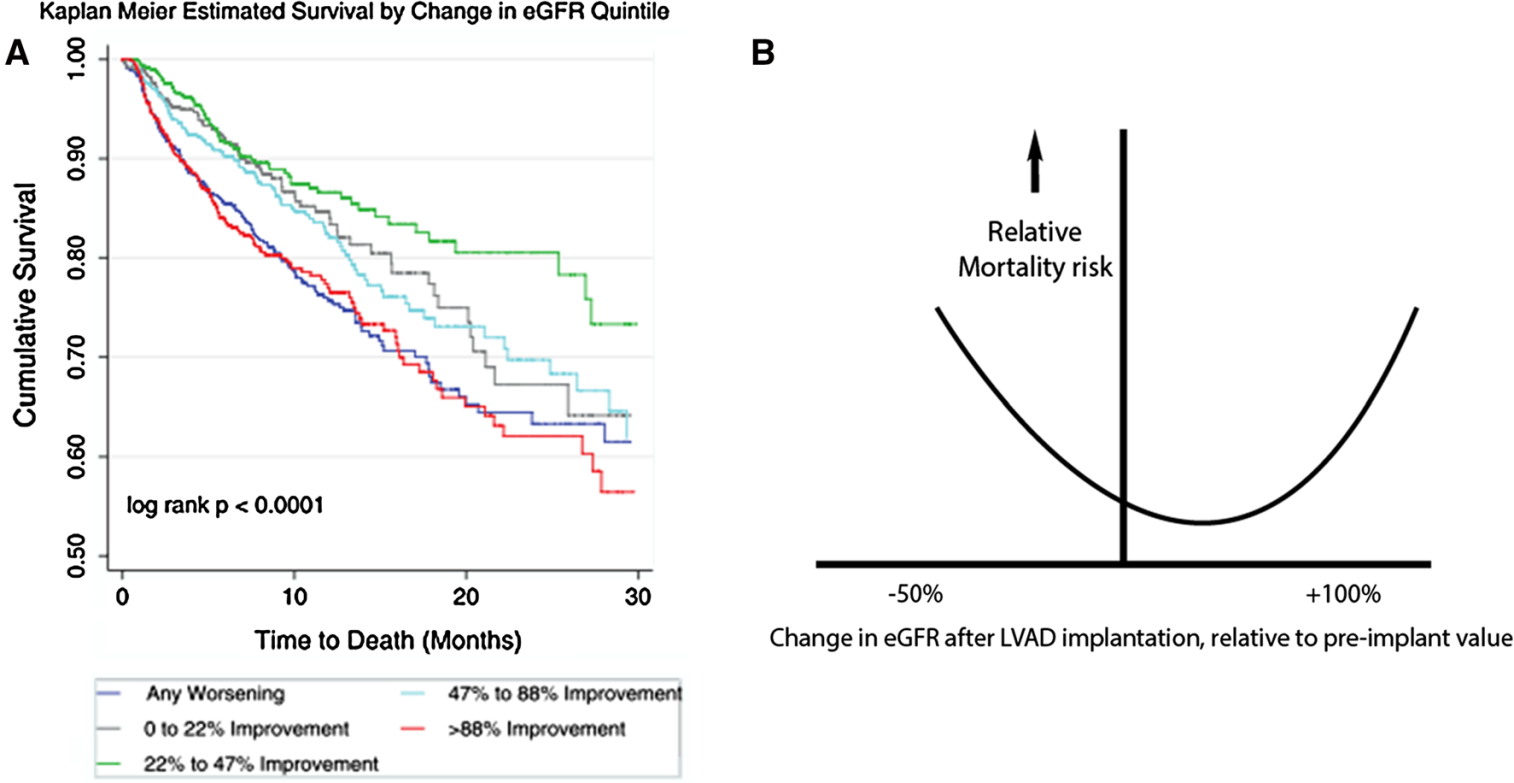
**a** (*Left*) Kaplan–Meier analysis of LVAD recipients grouped in changes in eGFR. Change in eGFR was taken from baseline to 1 month following surgery and represented as  % change (reprinted with permission [19] ). **b** (*Right*) schematic representation of effects of changes in early eGFR after LVAD implantation on relative mortality risk. The increased mortality risk on the left side of the U-curve is related to AKI. Surprisingly, large increases in eGFR are also associated with increased mortality risk [19]. The nadir of the U-curve lies toward a modest increase

Surprisingly, not only the rapid deterioration of renal function, but also a prompt increase in eGFR serves as a marker for increased mortality (Fig. 2a) [19]. Mortality prediction over changes in eGFR therefore appears to follow a U-curve (Fig. 2b). An explanation for this puzzling observation remains elusive. Perhaps relatively healthy patients who already have a near-normal eGFR can only regain a small proportion of their eGFR. These patients experience good survival. In contrast, very sick patients with severely compromised eGFR may initially experience as much as a doubling of eGFR. However, this cohort of very sick patients also experiences higher perioperative mortality.

## Patient selection

The fact that patients with preimplant RD have a higher mortality risk, combined with the fact that the majority of these patients nevertheless experience improvement in renal function after LVAD placement, creates a dilemma for the practicing physician concerning patient selection. Some have listed renal disease, marked by serum creatinine (sCr) ≥ 2.5 mg/dL or hemodialysis, as a relative contraindication for LVAD placement [38]. Kirklin et al. [16] show that dialysis prior to LVAD placement is accompanied with a 2.37 mortality hazard ratio. Nevertheless, others argue that patients with severe RD, even requiring renal replacement therapy (RRT) preimplant, need not be excluded from receiving LVADs [45–48]. Optimization of renal function prior to LVAD can be achieved by diuretics in an attempt to decrease renal venous congestion [38]. Not all HF patients have irreversible kidney damage; in fact, kidney function improves markedly early following LVAD placement. LVAD therapy has therefore been suggested as bridge to HTx candidacy: Improved circulation should restore renal function sufficiently to be eligible for HTx [49, 50]. Note that eligibility for LVAD therapy depends on many factors, of which renal function is only one [38, 51].

There are no definitive tests that can reliably predict reversibility of RD [52, 53]. Nevertheless, in the acutely decompensated HF population, Brisco et al. [53] show a linear relationship between BUN/creatinine ratio upon admittance, and percentage of patients that experience improved renal function (eGFR improved >20 %) upon return to cardiac compensation. This relationship has not been replicated in the LVAD population.

## Initial recovery of renal function

The short-term effects of LVAD therapy on renal function have been widely studied and well documented in various reviews [20, 49]. Renal function generally improves directly after LVAD implantation if decreased GFR is due to low perfusion before implantation [54]. Reduction in sCr from as high as 4.6–1.2 mg/dL has been observed [55]. Most of the recovery tends to occur in the first month after LVAD placement [56] with the most significant improvement occurring in the subpopulation with the most reduced preoperative renal function [56, 105]. Hasin et al. [57] found that 68 % of patients with a preimplant eGFR <60 mL/min showed an improvement in eGFR to above 60 mL/min after the first month. By analyzing the extensive INTERMACS database, Brisco et al. [19] reported a median improvement in eGFR of around 50 % by month 1, with 17 % of the LVAD population even doubling eGFR.

Improvement in renal function after LVAD implantation largely relies on reversal of several factors, both hemodynamic and non-hemodynamic, attributed to chronic CRS type II. The improvement in eGFR after LVAD implantation may in part be through improvement in intrarenal hemodynamics [58] and reversal of renal hypoperfusion [1]. The importance of the effect of hemodynamic factors is underscored by the observation that higher pump speeds at hospital discharge are associated with larger early increases in GFR [57]. It has been found that plasma renin activity and plasma aldosterone decrease significantly from baseline through weeks 4 and 8 following LVAD implantation [59]. This means that RAAS activation in HF is importantly reduced after LVAD implantation [60], providing biochemical confirmation of the improvement in hemodynamic status [49]. In addition, a decrease in sympathetic tone, as measured by renal sympathetic nerve activity, has been measured in animal models [61]. Reduced sympathetic nerve activity may be mediated by the aortic and cardiopulmonary baroreflex system and may lead to a decrease in renal vascular resistance [49]. In humans, plasma epinephrine and plasma norepinephrine levels also decrease after LVAD implantation [62].

In patients with near-normal preimplant renal function, these early improvements are less pronounced [63]. Butler et al. [52] found that the absence of diabetes was the only variable that could predict recovery of renal function post-LVAD, an observation which was confirmed [35]. However, a more recent study did not find diabetes to be a significant predictor of postimplant renal function improvement but indicated older age and smaller kidney size as negative predictors [57]. Brisco et al. [53] retrospectively studied reversibility of RD in a large number of decompensated HF patients and concluded that an elevated BUN/creatinine ratio upon admission could predict improvement of renal function with return to cardiac compensation. At the same time, recurrence of RD was common after discharge. These findings have yet to be replicated in the LVAD patient population.

## Renal function in the long run

Interestingly, it appears that no further improvement in renal function occurs from about 1 month after pump placement. In fact, many studies have observed a slow but gradual decrease in renal function several months postimplant [35, 52, 57, 64–67], although others failed to observe this phenomenon [56, 63, 68]. Table 2 shows the change in renal function after the period of initial recovery in various studies.

**Table 2:** Change in renal function after the initial period of recovery following placement of continuous-flow LVAD

Period (weeks)	Change in GFR	How GFR was estimated: mean change over the indicated period of observation	Participants (*N*)	References
2–12	↓	sCr: 0.8–1.0 mg/dL	43	Jacobs et al. [66]
4–12	↔	eGFR: 87 (±32)–90 (±31)	30	Kamdar et al. [69]
4–12	↓	eGFR: 87 (±28)–78 (±23)	83	Hasin et al. [57]
4–12	↓	eGFR: 84 (±33)–75 (±30)	55	Sandner et al. [70]
2–26	↑	eGFR: 62–74	116	Singh et al. [56]
4–26	↓	sCr: 1.0–1.1 mg/dL	126	Deo et al. [65]
4–26	↓	eGFR: 81 (±33)–63 (±25)	86	Sandner et al. [35]
4–26	↔	"…renal function showed improvements […] stabilizing by approximately 1–2 months of LVAD support with no further change afterward"	309	Russell et al. [63]
Discharge to 52	↓	eGFR: 96–71	27	Feitell et al. [67]
12–52	↓	sCr: 90–100 µmol/mL	85	Lok et al. [64]

*eGFR* estimated glomerular filtration rate (mL/min/1.73 m^2^), *NA* not available, *sCr* serum creatinine

**Fig. 3:**
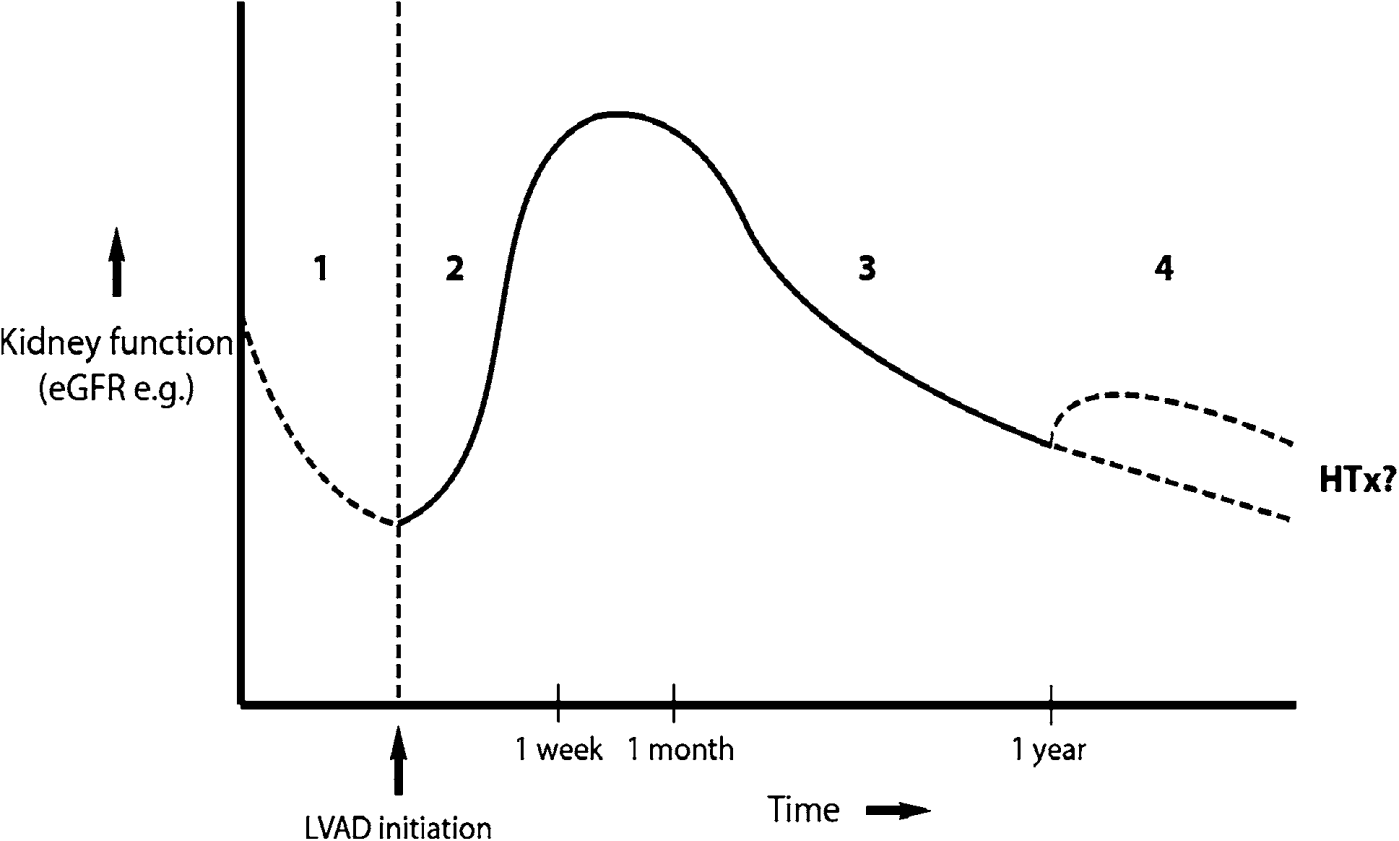
Schematic representation of evolution in renal function over time. *Phase 1* renal function declines with varying degrees as a result of CRS type II. *Phase 2* renal function initially recovers thanks to LVAD implantation and negation of renal hypoperfusion. This effect is most notable from several weeks to up to 2 months following implantation. *Phase 3* the functional improvement was only transient, and renal function continues to decline. Patients with the largest improvement consequently experience the largest deterioration, although, on average, the end-point renal function stays elevated over preimplant values, at least up to 1 year following transplantation. *Phase 4* hypothetically, in the long term, renal function continues to decline and may necessitate RRT (*lower dotted line*). Alternatively, the patient receives a heart transplantation, which can either temporarily alleviate the downward trend (*upper dotted line*) or leave it unaltered (*lower dotted line*)

Brisco et al. [19] analyzed 3,363 patients from the INTERMACS database and concluded that renal function improvement was transient. By 12 months, eGFR was only 6.7 % above the preimplant value. The changes in renal function over time have been schematically represented in Fig. 3.

Of special note are the (hypothetical) developments under phase 4 (dotted lines). The downward trend in GFR initiated after the initial 1- to 2-month peak may continue to decline in the long term. Unfortunately, sufficient reliable data for this time period are lacking. In addition, paired sample analyses combining the development of renal function following consecutive LVAD and HTx in the same patients are scant. Some research suggests that HTx following LVAD leaves the downward trend unaltered [71] although others report that HTx may temporarily increase GFR in patients with prior compromised renal function [72]. Singh et al. [56] retrospectively analyzed the evolution of renal function of 116 patients consecutively undergoing MCS and HTx. They reported a clear decrease in GFR following HTx, due to tacrolimus administration. However, renal outcomes after HTx seemed to be more dependent on the level of renal function achieved during MCS than on the level of renal function before MCS.

**Fig. 4:**
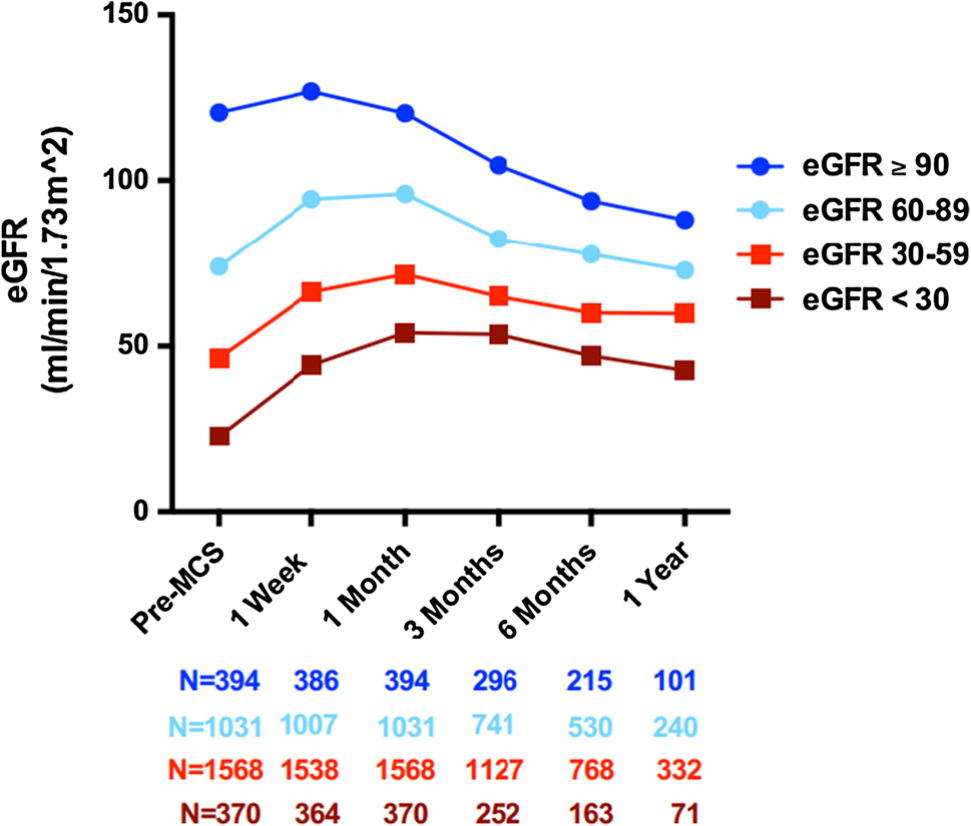
Change in eGFR over time, stratified by preimplant cohort, as reported by Brisco et al. [19] (reprinted with permission). Patients with low preimplant eGFR (*red lines*) appear to derive most benefit after MCS, with eGFR remaining notably elevated above preimplant levels up to 1-year after placement. By contrast, patients with moderate to good preimplant eGFR (*blue lines*) may undergo a net decrease in eGFR. *Note* that the fraction of patients with eGFR ≥ 90 mL/min is relatively small, and that the majority of patients have an eGFR < 60, as expected due to high prevalence of CRS type II in this population

Moreover, as shown in Fig. 4, changes in eGFR vary considerably depending on preimplant levels. Patients with eGFR ≤ 60 mL/min experience a net increase up to 1 year after implantation, whereas the eGFR of those patients with good preimplant values (dark blue line) may actually decrease after 1 year. Note that patients with eGFR ≥ 90 mL/min only represent a minority (12 %), and that the majority of patients have an eGFR ≤ 60 mL/min, due to the presence of CRS type II.

**Fig. 5:**
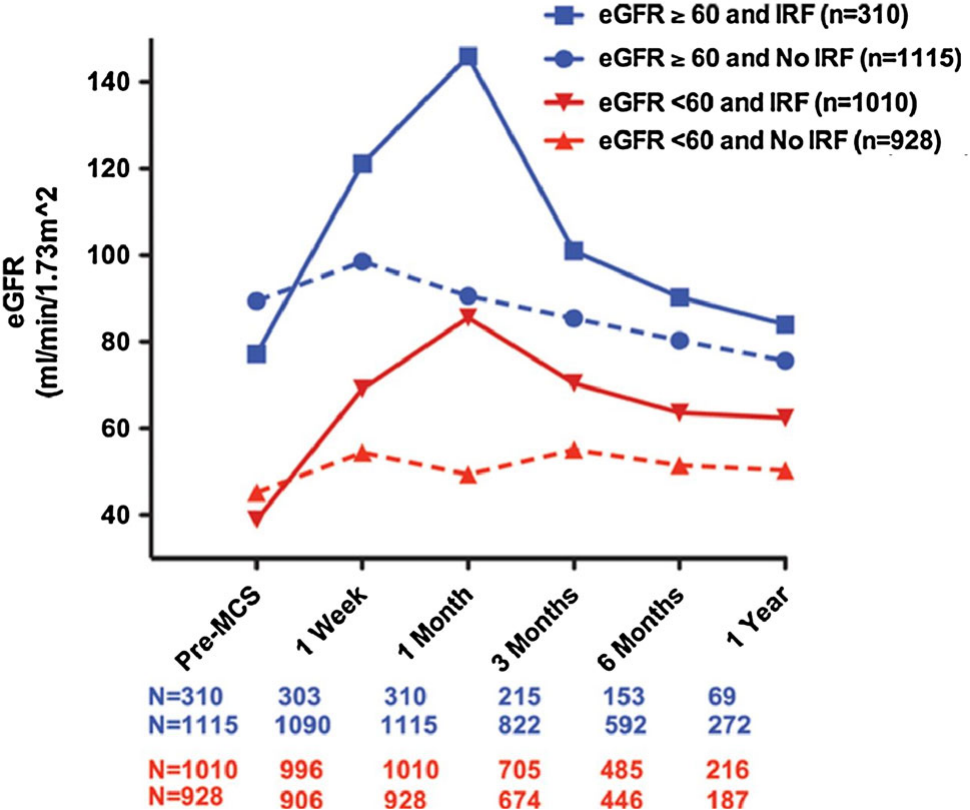
Changes in eGFR, first stratified by preimplant eGFR (*blue* and *red lines*), and subsequently divided between patients who experienced improved renal function (IRF, *solid lines*) and those who did not (no IRF, *dotted lines*). IRF is defined by an increase ≥ 50 % at month 1 over baseline renal function. Although the renal function quickly declined again after 1 month in the IRF group, the eGFR remained higher compared to the non-IRF group at 1 year post-implantation [19] (reprinted with permission). *Note* that the *dark blue solid line* surpasses an eGFR of 120 mL/min at month 1 (indicated by *horizontal red line*), a value that is considered above the normal range of GFR maintained by autoregulation. This may hint at ongoing hyperfiltration, which can lead to renal damage

Another interesting observation made by Brisco et al. is the fact that patients experiencing the largest increase in eGFR also have the largest subsequent deterioration. Nevertheless, this subpopulation still has a higher eGFR compared to the patients who did not significantly increase filtration after LVAD placement. The ‘volatile’ changes in eGFR displayed in Fig. 5 can provide further evidence for the transient nature of increased GFR directly after initiation of MCS. Note that the rate of GFR decline, on average, is much larger than that expected with age [73], CKD stage 3 [74] or even diabetic kidney disease [75] (declines of ±1, ±1 and ±3.5 mL/min/1.73 m^2^ per year, respectively). Several explanations for this trend have been proposed, which need not be mutually exclusive, but could in fact work synergistically.

### Measurement bias

One explanation for the trend in Fig. 3 is the measurement bias of sCr. End-stage HF patients are often bedridden and show signs of cachexia [57, 76]. Following LVAD placement, exercise capacity is restored, which might lead to an increase in muscle mass. Importantly, this might increase the sCr level, dependent on muscle mass [32]. Measuring GFR using cystatin C, a marker independent of muscle mass, might control for this bias. However, literature on this subject in the LVAD population is lacking.

Another explanation, proposed by Sandner et al. [35], is that patients lose the optimal medical care (particularly fluid balance management) they enjoyed during their hospital stay. Lack of improvement in renal function frequently corresponded with hospital discharge and may thus be attributed in part to inadequate patient self-management.

### Hemolysis

Another explanation for the late gradual decrease in renal function is that a sub-clinical level of hemolysis causes tubular damage [77]. Current commonly used devices operate at pump speeds approaching 10,000 and 3,000 rotations per minute (axial- and centrifugal flow, respectively) [78]. Erythrocytes can lyse under high shear stress, created either through pump speed or partial pump thrombosis. Thrombotic plaques may obstruct the inflow cannula and change blood flow patterns in favor of non-laminar flow. This puts erythrocytes under increased shear stress, causing hemolysis which can ultimately lead to kidney injury [79]. Free iron from hemolytic cells can initiate inflammation around the nephrons [77] and free hemoglobin could precipitate with Tamm-Horsfall and cause intratubular obstruction [80]. Moreover, the free hemoglobin could decrease the availability of nitric oxide, leading to renal vasoconstriction and ischemia [77].

### Right-sided HF

A serious complication after prolonged LVAD support is right-sided HF [81, 82]. A recent post-market evaluation study showed right HF to occur in 9 % of patients, representing 0.10 events per patient-year [83]. Left ventricular unloading by the LVAD may promptly increase venous return and overload the right ventricle (RV). Increased filling pressure may cause RV overdistension and decrease myocardial perfusion, leading to RV failure. This, in turn, results in venous congestion in the kidneys (and other organs) [82], reducing perfusion and eGFR [27].

### Pulsatility

The non-physiological nature of reduced pulsatility in cf-devices has been reported as a possible factor for decreasing renal function [84]. In humans, insufficient data exist on effects of reduced pulsatility on long-term end-organ function, although functions are maintained within normal range for up to 15 months [85]. Surprisingly, analysis of INTERMACS data reveals that gradual late decline in eGFR was observed with both cf-LVADs and pf-LVADs, hinting that gradually declining kidney function cannot solely be attributed to reduced pulsatility [19]. Some studies found no detrimental effects or major differences in renal function comparing cf-LVAD to pf-LVAD patients [19, 63, 86].

Nevertheless, reduced pulsatility most likely induces profound morphological changes in the large vasculature. Animal [87] and human [88, 89] studies have shown that prolonged continuous flow caused significant changes in the aortic wall, including medial degeneration, smooth muscle cell (SMC) disorientation and depletion, elastic fiber fragmentation and depletion, medial fibrosis and atherosclerotic changes [89]. Such changes may translate to decreased peripheral vascular reactivity [90, 91]. It has been suggested that continuous flow leads to stiff and unresponsive arteries [92].

Animal studies report proliferation of SMCs in the afferent arteriole in the renal cortex [93, 94] and perivascular tissue [94], but could not determine whether this change in morphology affected afferent arteriolar constriction and renal function. Infiltration of inflammatory cells in the renal cortical matrix has been observed, suggesting an immunologic mechanism for SMC hypertrophy [93]. Interestingly, reduced pulsatility may induce (severe) peri-arteritis in the kidneys, an observation not made in control animals supported by pf-devices. Peri-arteritis has been linked to upregulation of the local RAS system [93]. Blood contact with the device’s artificial surface may activate inflammatory cells to induce inflammation [95, 96], although this should be largely prevented by formation of a pseudo-intima in the LVAD [78].

Latif et al. studied the GFR of LVAD-bridged and medication-bridged HTx recipients. Despite near identical patient characteristics and renal function at baseline, the medication-bridged population showed a higher GFR compared to the LVAD-bridged group following HTx [71]. This suggests that the LVAD may induce some permanent structural damage in the kidney.

Arguably, the late and gradual decline in renal function does not pose the largest clinical challenge for the duration of support at this point. For most patients, renal function can be maintained within normal ranges for the duration of support. However, with a growing number of DT patients, and the fact that LVADs are to be used for significantly longer periods of time, it is of the utmost importance to ascertain the causes of decreasing renal function. Because durability of MCS has improved, the cumulative effects of long-term support on non-cardiac organ function have become an important topic [19]. Introduction of pulsatility in rotary blood pumps [85] and reduction in shear stress may be necessary to further improve results [17].

## Acute kidney injury after LVAD

Acute kidney injury (AKI) is one of the major perioperative adverse events following LVAD placement. Table 3 summarizes the incidence of AKI in cf-LVAD devices. Patel et al. [20] and Mao et al. [49] also reviewed AKI in the LVAD population. AKI is defined as a doubling of baseline sCr level or reduction of eGFR by 50 % following LVAD implantation [33]. Note that patient characteristics at baseline can greatly influence the occurrence of postimplant AKI. The incidence of AKI after cf-LVAD implantation varies considerably: between 4 and 38 % (see Table 3). It appears that studies from the early era of MCS report higher incidences of AKI compared to the most recent post-market evaluation studies. Reasons for this improvement are uncertain, but may include better patient selection and increased surgical experience.

**Table 3:** Incidence of acute kidney injury after implantation of continuous-flow LVAD

Enrollment period	Incidence AKI (%)	EPPY	LVAD type	Definition of AKI	Patients at baseline	References	Notes
9/1994–1/2007	24/63 (38)	NA	DeBakey VAD (59) HVAD (2) Terumo DuraHeart LVAD (2)	AKI = RRT	sCr 1.4 (±0.6)	Sandner et al. [70]^a^	
11/1998–7/2007	30/86 (35)	NA	DeBakey VAD (75) HVAD (6) DuraHeart LVAD (5)	AKI = RRT	sCr 1.3	Sandner et al. [35]	
9/2002 –8/2005	4/14 (29)	NA	Jarvik 2000	AKI = RRT	sCr 1.5 (±0.5)	Feller et al. [103]^a^	
11/2003–6/2009	15/107 (14)	NA	HMII	AKI = RRT	sCr 1.9 (±0.6)	Demirozu et al. [45]	^b^
3/2005–5/2006	18/133 (14)	0.31	HMII	ND	eGFR 75 (±37) All NYHA IV	Miller et al. [104]	
3/2005–5/2007	21/133 (16)	0.10	HMII	ND	sCr 1.6 (±0.6) 71 % NYHA IV	Slaughter et al. [105]	
3/2005–4/2008	30/281 (11)	0.17	HMII	ND	eGFR 79 (±35) All NYHA IV	Pagani et al. [106]	
3/2006–12/2008	5/50 (10)	0.10	HVAD	ND	sCr 1.3 (±0.5) Intermacs profile II (22 %) III (78 %) IV (8 %)	Strueber et al. [107]	
3/2006–7/2011	28/100 (28)	NA	HMII and HVAD	RIFLE stage II and greater	sCr 1.4	Borgi et al. [98]	^c^
3/2006–12/2011	9/85 (11)	0.08	HMII	AKI = RRT	sCr 120 μmol/l Intermacs profile I (25 %) II (75 %)	Lok et al. [64]	
2/2007–6/2010	8/83 (10)	NA	HMII	AKI = RRT	sCr 1.6 (±0.7) 62 % NYHA IV	Hasin et al. [57]	^d^
5/2007–3/2009	30/281 (11)	0.06	HMII	ND	sCr 1.5 (±0.6) 63 % NYHA IV	Park et al. [111]	^e^
4/2008–8/2008	17/169 (10)	0.13	HMII	ND	sCr 1.3 (±0.5) Intermacs profile I (24 %) II (37 %) III–VII (39 %)	Starling et al. [112]	
4/2008–10/2010	129/1496 (9)	0.14	HMII	ND	sCr 1.4 (±0.8) Intermacs profile I (17 %) II (45 %) III–VII (38 %)	John et al. [109]	
8/2008–8/2010	12/140 (9)	0.16	HVAD	ND	sCr 1.3 (±0.4) Intermacs profiles I (5 %) II (24 %) III (52 %) IV–VII (19 %)	Aaronson et al. [108]	
8/2008–7/2012	32/332 (10)	0.13	HVAD	ND	eGFR 87 (±39) 96 % NYHA IV Intermacs profile I (6 %) II (40 %) III (42 %)	Slaughter et al. [110]	^f^
2/2009–11/2012	10/254 (4)	0.04	HVAD	ND	sCr: ND NYHA: ND	Strueber et al. [83]	

Some studies repeat results of previous publications. Care was taken to disentangle those results and only represent the ‘new’ patients, not previously published

*AKI* acute kidney injury, *BTT* bridge to transplantation, *DT* destination therapy, *eGFR* estimated glomerular filtration rate (mL/min/1.73 m^2^), *EPPY* events per patient-year, *HMII* HeartMate II (Thoratec Inc., Pleasanton, CA), *HVAD* HeartWare ventricular assist device (HeartWare Inc., Framingham, MA), *Intermacs* Interagency Registry for Mechanically Assisted Circulatory Support, *LVAD* left ventricular assist device, *NA* not available, *ND* not defined, *NYHA* New York Heart Association, *RRT* renal replacement therapy, *sCr* serum creatinine (mg/dL)

^a^These studies included both pulsatile and continuous-flow devices. However, outcomes of pf-LVADs were omitted

^b^This single-center study only included patients supported for more than 30 days

^c^32 % of patients included in this study received LVAD as destination therapy

^d^68 % of patients included in this study received LVAD as destination therapy

^e^All of the patients included in this study received LVAD as destination therapy

^f^140 patients included in this study were already previously reported by Aaronson et al. [108]

AKI is clearly a negative survival predictor [97]: patients experiencing AKI after LVAD implantation generally have a longer length of hospital stay and increased 30-day mortality [39, 98]. Genovese et al. predicted a threefold increased risk for 1-year mortality in case of AKI [99]. Several mechanisms of postoperative AKI, both functional and histological, have been proposed. Device routing may predispose hemodynamic instability, enhance thrombogenicity and spawn small emboli to the kidney, which have been observed as small renal infarctions in a lamb model [100]. In addition, cardiopulmonary bypass time [39, 101] and number of blood transfusions [77, 102] have been linked to AKI. Alternatively, hemolysis induced by high shear stress from the rotor, releasing free hemoglobin and iron, could play a role [77, 79]. These effects may act synergistically.

## Renal replacement therapy

A variable number of LVAD recipients experiencing AKI after LVAD placement may require renal replacement therapy (RRT). In a recent Dutch study, 11 % of patients required post-surgical continuous venovenous hemofiltration [64], but this number may even reach 33 % [45, 97, 113]. Fortunately, the majority of patients recover from AKI and RRT can in many cases be discontinued after about 1 month [114].

Patients with preimplant RRT are only sporadically admitted for LVAD treatment; about 1.5 % of all new patients required dialysis before LVAD implantation [16]. This subpopulation is at increased mortality risk [16, 33], which makes clinicians more hesitant to initiate LVAD therapy. Nevertheless, successful results have been obtained and some of these patients could be weaned off RRT [45]. There is anecdotal evidence of patients whose renal function failed to improve either due to irreversible renal damage or early mortality [45, 115]. Incidentally, patients stay on RRT during LVAD support before combined heart and kidney transplantation [45].

Theoretically, some LVAD patients will experience gradual progression of CKD. If this gradual decline continues steadily and over a significant period of time, it will ultimately necessitate RRT. Considering the fact that widespread use of DT has only recently taken root, it is too early to evaluate if this subpopulation of RRT LVAD patients will occur.

### Hemodialysis versus peritoneal dialysis

There are two options for RRT in the LVAD population, hemodialysis (HD) or peritoneal dialysis (PD). HD is currently the default option [6], although it has been suggested to give more consideration to PD because of its decreased chance of systemic infection [115, 116] and hemodynamic instability. Cross-contamination with the driveline site can be minimized by placing the PD catheter as far away as possible from the driveline exit site [116]. However, this option is not available for devices that need to be placed sub-diaphragmatically. Results with PD in LVAD patients are encouraging [116], but due to a lack of randomized controlled prospective trials, superiority over HD cannot yet be claimed [117].

### Management challenges

AKI necessitating RRT poses considerable challenges [117]. Hemodynamic instability and the fact that LVAD patients show reduced to no pulse makes continuous pulse and BP monitoring difficult. Normal arm-cuff BP measurements are less reliable. Instead, blood flow and BP can be estimated using Doppler probe devices [117]. This was shown to be successful in over 90 % of attempts, roughly twofold the success rate of arm-cuff measurements [118].

Theoretically, there are several dangers of HD for LVAD patients. Currently, cf-LVADs are volume-sensitive and can malfunction due to intravascular fluid flux inherent to HD [114]. Reduced blood volume as well as increased pump speed can create a so-called suction event in which the left atrium and ventricle collapse [114, 117]. Changes in pump speed due to volume sensitivity during HD can also induce thrombosis (reduced speed) or hemolysis (increased speed) [114, 117]. Although challenging and not without risks, HD can be given to LVAD patients safely under specialized supervision. A recent report showed that only 5 % of HD sessions were interrupted or terminated, with no serious adverse effects noted [114].

## Future outlook

In the USA, the annual number of LVAD placements (>2000 [16]) has surpassed the number of heart transplantations since 2009 [119]. In 2011, 37 % of HTx recipients were bridged to transplantation with an LVAD [120], and this percentage is steadily increasing [121]. Currently, DT accounts for over 40 % of new implants [16], and this fraction is also expected to grow [84]. In Europe, LVADs are still predominantly used for BTT, although the tide may shift toward DT in the (near) future, mimicking the trend abroad. In Japan, which faces an extremely low availability of donor hearts, DT has already been proposed as the new gold standard for treatment of end-stage HF [122].

Several developments can accelerate the growing acceptance of implantable MCS devices for DT, including device miniaturization, development of less invasive surgical techniques and reduction of adverse event burden [123]. At present, the percutaneous driveline cable considerably limits patients’ quality of life and poses risk for infection. Transcutaneous energy transfer systems [124] and, alternatively, free-range resonant electrical delivery [125] will render driveline cables obsolete. Successful implementation of transcutaneous energy transfer systems will mark a decisive turning point for the use of LVAD technology in mainstream therapy of advanced HF [126]. However, documented reduction in adverse event burden is urgently required before a paradigm shift of MCS as true alternative to HTx can occur [123].

At present, there is not enough evidence to support the durability and reliability of LVAD therapy for lengths of time comparable to HTx. There are no reliable data concerning end-organ function after prolonged LVAD support (e.g., >5 years) [33], although it is expected that 10-year survival can soon be achieved with current devices [37]. Currently, only around 100 LVAD patients have survived longer than 5–7.5 years [17, 48]. Long-term studies (>1 year) are needed to assess effects on end-organ function with continuous-flow devices, which may have important implications for use as DT [69, 127]. Gradually declining renal function may have important clinical consequences if LVADs are expected to offer long-term chronic support as DT. The transient nature of renal recovery also has relevance to the BTT population. Haglund et al. [128] recently demonstrated that LVAD patients with pre-HTx GFR < 45 mL/min/m^2^ show reduced graft survival after HTx.

There are many research avenues to be investigated. The extent to which declining GFR could be attributed to sCr measurements biased for muscle mass can be investigated using a muscle-mass independent serum marker such as Cystatin C. In addition, possible reduction of in vivo erythrocyte survival should be investigated to ascertain the possible role of subclinical hemolysis and consequent nephrotoxicity. Closer attention can be paid to the long-term effects of continuous-flow support, particularly in relation to renal (micro) vasculature. Better understanding of the relationship between the LVAD and the kidney may aid development of more durable devices and help improve patient selection.

## Conclusion

In conclusion, LVAD therapy has become an established treatment option for end-stage HF patients. Although difficult to predict, following LVAD implantation CRS type II is often relieved quickly in this population. Interestingly, early recovery appears to be transient and is followed by a gradual decline in GFR starting 1–2 months following implantation. Larger increases in GFR are followed by a proportionally larger decline later on, although GFR generally remains above preimplant levels for duration of follow-up. LVAD patient outcomes continue to improve, and adverse events including AKI are on the decline. Emerging technological advances such as transcutaneous energy transfer are expected to greatly improve quality of life in the near future and may allow these devices to start rivalling HTx. However, considering the growing acceptance of DT, it is of the utmost importance to be informed on long-term cumulative effects of the LVAD on the kidneys. Additional experience, gained with both research and passage of time, is required to further unravel the intricate relationship between LVADs and the kidney.
